# Predictors of failed attendances in a multi-specialty outpatient centre using electronic databases

**DOI:** 10.1186/1472-6963-5-51

**Published:** 2005-08-06

**Authors:** Vernon J Lee, Arul Earnest, Mark I Chen, Bala Krishnan

**Affiliations:** 1 Department of Clinical Epidemiology, Tan Tock Seng Hospital, Singapore; 2 Division of Operations, Tan Tock Seng Hospital, Singapore

## Abstract

**Background:**

Failure to keep outpatient medical appointments results in inefficiencies and costs. The objective of this study is to show the factors in an existing electronic database that affect failed appointments and to develop a predictive probability model to increase the effectiveness of interventions.

**Methods:**

A retrospective study was conducted on outpatient clinic attendances at Tan Tock Seng Hospital, Singapore from 2000 to 2004. 22864 patients were randomly sampled for analysis. The outcome measure was failed outpatient appointments according to each patient's latest appointment.

**Results:**

Failures comprised of 21% of all appointments and 39% when using the patients' latest appointment. Using odds ratios from the mutliple logistic regression analysis, age group (0.75 to 0.84 for groups above 40 years compared to below 20 years), race (1.48 for Malays, 1.61 for Indians compared to Chinese), days from scheduling to appointment (2.38 for more than 21 days compared to less than 7 days), previous failed appointments (1.79 for more than 60% failures and 4.38 for no previous appointments, compared with less than 20% failures), provision of cell phone number (0.10 for providing numbers compared to otherwise) and distance from hospital (1.14 for more than 14 km compared to less than 6 km) were significantly associated with failed appointments. The predicted probability model's diagnostic accuracy to predict failures is more than 80%.

**Conclusion:**

A few key variables have shown to adequately account for and predict failed appointments using existing electronic databases. These can be used to develop integrative technological solutions in the outpatient clinic.

## Background

Failure to comply with outpatient medical appointments is a perennial problem, affecting costs, causing scheduling conflicts, and interrupting continuity of care. Failed appointments in different outpatient settings have ranged from 12% to 42% [1-7]. The resulting economic costs range from £65 per failed appointment in the United Kingdom in 1997 [2] to 3–14% of total outpatient clinic income in the United States [8]. This problem may be compounded if non-compliance with appointments is an indication of poorer clinical outcomes [9]. Most studies on failed appointments focused on the socio-economic and demographic factors that affect failures [1,10-13]. Other factors studied include symptom duration or resolution, illness, long waiting periods, forgotten appointments, and other commitments [13-16]. Successful interventions have included reminders, giving the patient's choice of date, improved communication, and selective overbooking [2,10,17]. However, almost all studies were for specific specialties in small-scaled settings [2,5,8-13].

We wanted to determine the intrinsic and external factors affecting failed outpatient appointments using only routinely available data. Our objective was to examine the factors most associated with failed appointments in Singapore, and to devise a prognostic index that administrators may use to identify potential defaulters. The findings will allow administrators to account for these factors when scheduling attendances, and provide the platform for problem solving. Such a prognostic index will also allow targeting of patients at higher risk of defaulting hence reducing the costs of intervening in patients who do keep their appointment.

## Methods

This was a retrospective cohort study on patients attending all outpatient clinics at Tan Tock Seng Hospital, a 1400 bed general hospital in Singapore. Data was obtained from the hospital's appointment systems database and included 3,212,789 outpatient appointments starting from the creation of the electronic database in August 2000, to July 2004. Cancelled or rescheduled appointments were excluded, and a computer generated random sample of 10% of patients was used.

### Outcome measures and input factors

The outcome measure was failure of a patient to attend his most recent appointment, analysed for individual patients who had at least one visit from August 2001 to July 2004. This allowed us to have at least one year of appointment history (starting August 2000) for all patients.

A system-unique alphanumeric patient identifier was then used to sort all appointments by individual patients. The most recent appointment was then selected and coded as "actualised" if the patient registered during the scheduled clinic session, or "failure" if the patient did not attend the appointment. The same process was used to identify the appointment history for each patient. To account for the varying frequency and duration of follow-up between patients, we analysed past history of failed appointments as a proportion of all scheduled appointments, hence allowing us to use the entire database for the predicted probability model. Patients with no record of previous appointments within the entire database period starting August 2000 were classified separately. As the maximum inter-appointment duration is usually not longer than a year, we could assume that cases seen after August 2001 with no prior database records were correctly classified as having no prior appointments.

Other factors studied included the patient's gender, race, age-group, days from scheduling to appointment, percentage of previous appointment failures, provision of cell phone numbers, distance from place of residence, and hospital admission during the appointment or between scheduling and appointment. Reasons for failed appointments were not obtained as there was no routine provision for contacting patients who defaulted. Direct distance from the patient's residence to the hospital was computed from the address zip codes and categorised into 3 groups – less than 6 km (1–2 districts away), 6 to 14 km (3–4 districts away), and more than 14 km (outlying districts). The data was stratified by specialties by categorising all 47 sub-specialty departments into 6 functional groups – medical subspecialties, surgical departments, ear, nose, and throat (ENT), ophthalmology, therapy, and others.

### Statistical methods

Data extraction and management was done in Microsoft Access and data analysis was performed using Stata [18]. All tests were conducted at the 5% level of significance and we reported the odds ratios and corresponding 95% confidence intervals.

We started with a univariate analysis on all variables by simple regression. As the effect of confounding has been previously shown to be important [19], multivariate analysis with a multiple logistic regression model was also performed starting from the most significant variable in the univariate analysis and adding the next most significant, using the likelihood ratio test to observe improvements in the model's fit. The coefficients from the logistic regression were used to formulate the predicted probability model. For the final model, we used a receiver-operating characteristic (ROC) curve to assess the model's discriminatory ability for appointment actualisation. The data was then stratified by the six specialty functional groups, and the final multiple logistic regression analysis repeated to observe for possible differences across specialty departments.

## Results

Failed appointments accounted for 21% of all appointments in the database. From our sampling, a total of 22864 patients were included and of the most recent visit for individual patients, 39% of these appointments resulted in failures. Table 1 gives the characteristics of the study population. 26% had no previous appointment record and more than 40% of appointments were in excess of three weeks after scheduling. Only a small proportion were actually hospitalised prior to, or during the appointment date (2% and 1% respectively). The majority of patients (60%) provided a cell phone number.

**Table 1:** Demographic characteristics and univariate factors associated with failed appointments, with the corresponding number of subjects (n), odds ratios, confidence intervals, and p-values (overall n = 22864).

**Variable**	**n (%) **	**OR **	**95% CI**	**p-value**
Gender				
Male	12453 (54%)	1		
Female	10411 (46%)	0.94	(0.90, 0.99)	0.035
				
Race				
Chinese	16951 (74%)	1		
Malay	2073 (9%)	1.51	(1.38, 1.66)	<0.001
Indian	2120 (9%)	1.73	(1.58, 1.90)	<0.001
Others	1715 (8%)	1.42	(1.29, 1.57)	<0.001
				
Age group				
Up to 20 years	2002 (9%)	1		
21 to 30 years	4298 (19%)	0.99	(0.89, 1.10)	0.838
31 to 40 years	4190 (18%)	0.97	(0.87, 1.08)	0.621
41 to 50 years	3992 (17%)	0.76	(0.68, 0.84)	<0.001
51 to 60 years	3265 (14%)	0.67	(0.60, 0.75)	<0.001
More than 60 years	5137 (22%)	0.75	(0.68, 0.84)	<0.001
				
Days from scheduling to actual appointment				
Up to 7 days	5852 (26%)	1		
7 to 21 days	7234 (32%)	1.05	(0.98, 1.13)	0.144
More than 21 days	9840 (43%)	1.24	(1.16, 1.33)	<0.001
				
Percentage of previous failed appointments				
Up to 20%	5288 (23%)	1		
21% to 40%	3584 (16%)	1.14	(1.04, 1.25)	0.007
41% to 60%	3596 (16%)	1.41	(1.29, 1.55)	<0.001
More than 60%	4414 (19%)	1.95	(1.79, 2.13)	<0.001
No previous appointment	6044 (26%)	4.67	(4.31, 5.06)	<0.001
				
Provided cell phone number	13813 (60%)	0.09	(0.09, 0.10)	<0.001
				
Approximate distance from TTSH				
<6 km	8114 (37%)	1		
6 to 14 km	8427 (39%)	0.99	(0.93, 1.06)	0.844
>14 km	5248 (24%)	1.11	(1.04, 1.20)	0.003
				
Admitted				
During appointment date	182 (1%)	0.86	(0.63, 1.16)	0.320
Between appointment scheduling date and actual appointment date	423 (2%)	0.87	(0.71, 1.07)	0.183
				
Department				
Surgical	7961 (37%)	1		
Medical	6848 (32%)	1.06	(0.99, 1.13)	0.106
ENT	1935 (9%)	1.14	(1.03, 1.27)	0.014
Ophthalmology	3721 (17%)	1.13	(1.04, 1.23)	0.004
Therapy	613 (3%)	2.64	(2.23, 3.11)	<0.001
Others	574 (3%)	13.4	(10.49, 17.11)	<0.001

### Analysis

In the univariate analysis (Table 1), we found that gender, race, age group, days from scheduling to appointment, previous failed appointments, provision of cell phone number, distance from the hospital, and department were all significantly associated with failed appointments.

From the multiple logistic regression analysis (Table 2), age group, days from scheduling to appointment, previous failed appointments, provision of cell phone number, distance from hospital, and department were independently and significantly associated with failed appointments. Those older than 40 years had significantly lower odds of appointment failure than those below 20. Malays and Indians had significantly higher odds ratio (OR 1.48 and 1.61 respectively) compared to Chinese. Scheduling to appointment time was a good predictor, and longer times increased the likelihood of failure (OR 1.29 for 7 to 21 days, and 2.38 for more than 21 days). Prior appointment history was also strongly predictive of failure. Patients with more than 40% failed appointments had significantly higher odds compared to those with less than 20%. Patients without previous appointments had the highest odds ratio of 4.38. Those residing more than 14 km from the hospital had a significant odds of failure 1.14 times that of those residing less than 7 km away. Those providing cell phone numbers were least likely to have failed appointments, with an odds ratio of 0.10 (95% CI: 0.10–0.11). Compared to surgical appointments, ENT, ophthalmology, therapy, and others had significantly higher odds of failure. Variables which did not improve the model's fit were gender, and hospital admission during or prior to appointment.

**Table 2:** Multivariate factors associated with failed appointments with the corresponding odds ratios, confidence intervals, and p-values.

Variable*	**OR**	**95% CI**	**p-value**
Age group			
Up to 20 years	1		
21 to 30 years (x_1_)	0.96	(0.83, 1.11)	0.575
31 to 40 years (x_2_)	0.93	(0.81, 1.08)	0.335
41 to 50 years (x_3_)	0.75	(0.64, 0.86)	<0.001
51 to 60 years (x_4_)	0.66	(0.57, 0.77)	<0.001
More than 60 years (x_5_)	0.84	(0.73, 0.97)	0.019
			
Race			
Chinese	1		
Malay (x_6_)	1.48	(1.31, 1.68)	<0.001
Indian (x_7_)	1.61	(1.42, 1.81)	<0.001
Others (x_8_)	1.03	(0.89, 1.18)	0.716
			
Days from scheduling to actual appointment			
Up to 7 days	1		
7 to 21 days (x_9_)	1.29	(1.16, 1.42)	<0.001
More than 21 days (x_10_)	2.38	(2.16, 2.62)	<0.001
			
Percentage of previous failed appointments			
Up to 20%	1		
21% to 40% (x_11_)	0.96	(0.85, 1.09)	0.565
41% to 60% (x_12_)	1.21	(1.07, 1.36)	<0.001
More than 60% (x_13_)	1.79	(1.60, 2.00)	<0.001
No previous appointment (x_14_)	4.38	(3.95, 4.86)	<0.001
			
Provided cell phone number (x_15_)	0.10	(0.09, 0.11)	<0.001
			
Approximate distance from TTSH			
<6 km	1		
6 to 14 km (x_16_)	1.02	(0.94, 1.11)	0.596
>14 km (x_17_)	1.14	(1.04, 1.25)	<0.001
			
Department			
Surgical	1		
Medical (x_18_)	0.92	(0.84, 0.99)	0.049
ENT (x_19_)	1.2	(1.05, 1.37)	0.008
Ophthalmology (x_20_)	1.13	(1.02, 1.26)	0.022
Therapy (x_21_)	4.73	(3.85, 5.82)	<0.001
Others (x_22_)	20.22	(15.34, 26.65)	<0.001

**Figure 1:**
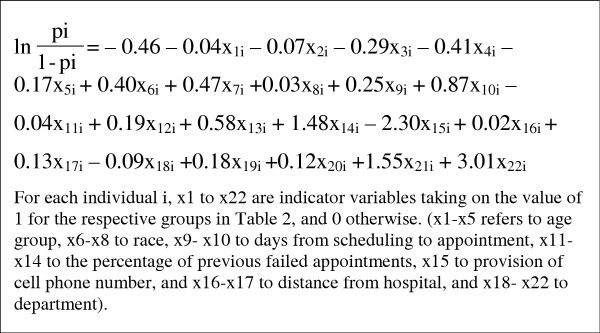
Predicted probability equation for appointment failure derived from the multiple logistic regression model.

* The indicator variables as used in the predicted probability equation in Figure 1 are shown in brackets () next to the respective variables.

### Predicted probability model

Based on the final model, we created a prognostic index to predict failed appointments. The predicted probability of failure (p_i_) was calculated using the equation shown in Figure 1.

**Figure 2:**
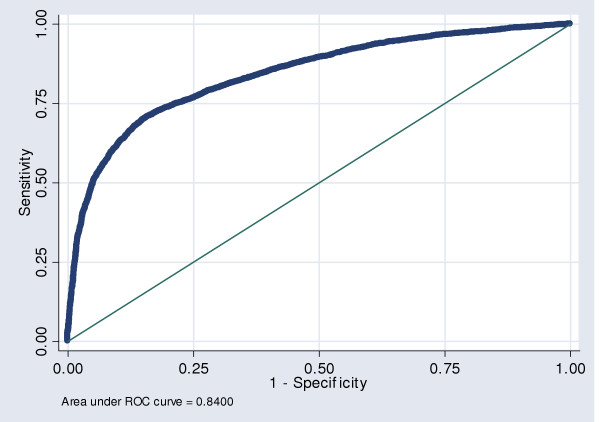
Receiver-operating characteristic curve of the final multiple logistic regression model for failed appointments.

From the final model's receiver-operating characteristic curve (Figure 2), the area under the curve of 0.84 (95%CI: 0.83–0.85) indicates that the model's overall diagnostic accuracy in predicting failed appointments is good. Using a cut-off of p = 0.24, the model had a sensitivity of 80%, specificity of 70%, and an accuracy of 73%.

### Stratification by department

We also performed a stratified analysis of the final multivariate model for department groups (Table 3). Provision of cell phone numbers was the only factor negatively associated with failed appointments across all departments, while no previous appointments was positively associated throughout. More than 21 days from scheduling to appointment was positively associated for all departments except therapy, where there was an insignificant negative association. Patients older than 40 years were negatively correlated with failed appointments except for elderly ophthalmology patients.

**Table 3:** Stratified analysis of factors by key departments

**Variable**	**Surgical (n = 7961)**	**Medical (n = 6848)**	**ENT (n = 1935)**	**Ophthalmology (n = 3721)**	**Therapy (n = 613)**	**Others (n = 574)**
Age group						
Up to 20 years	*1*	*1*	*1*	*1*	*1*	*1*
21 to 30 years	0.77*	0.99	0.88	1.36	1.12	0.87
31 to 40 years	0.85	0.81	0.76	1.25	1.12	1.23
41 to 50 years	0.71*	0.7*	0.52*	0.87	0.88	0.81
51 to 60 years	0.71*	0.66*	0.49*	0.62*	0.6	0.42
More than 60 years	0.89	0.69*	0.86	1.1	0.62	0.79
						
Race						
Chinese	*1*	*1*	*1*	*1*	*1*	*1*
Malay	1.69*	1.53*	1.26	1.35	0.98	0.91
Indian	1.44*	1.67*	1.53	1.85*	1.11	5.28*
Others	1.01	0.95	1.19	1.17	0.85	0.7
						
Days from scheduling to actual appointment						
Up to 7 days	*1*	*1*	*1*	*1*	*1*	*1*
7 to 21 days	1.46*	1.36*	1.1	0.95	2.07*	0.78
More than 21 days	2.77*	2.7*	2.28*	1.99*	0.64	2.8*
						
Percentage of previous failed appointments						
Up to 20%	*1*	*1*	*1*	*1*	*1*	*1*
21% to 40%	0.7*	1.37*	0.49*	0.97	1.24	1.18
41% to 60%	0.86	1.7*	1.06	1.21	1.98*	1.06
More than 60%	1.26*	3.1*	1.39	1.41*	1.59	3.25*
No previous appointment	3.84*	5.96*	4.07*	3.24*	1.86	4.37*
						
Provided cell phone number	0.07*	0.1*	0.06*	0.17*	0.15*	0.15*
						
Approximate distance from TTSH						
<6 km	*1*	*1*	*1*	*1*	*1*	*1*
6 to 14 km	1.04	1.06	0.81	1.07	1.02	1.07
>14 km	1.13	1.12	0.86	1.34*	0.98	1.46

Numbers presented within the table represent adjusted odds ratios. The baseline comparison group has an odds ratio of 1 and is italicised. Odds ratios significant at a level of p < 0.05 are indicated with an asterisk "*".

## Discussion

This study demonstrates that routinely available administrative data can be used to construct a prognostic index for appointment failures. Using a cut-off probability of above 0.24, the model identified defaulters with 80% certainty. Using the same cut-off, 30% of those who actualise their appointments would be wrongly classified. While imperfect, the model enables administrators to predict failed appointments with reasonable certainty for targeted intervention. Interventions have been shown to improve attendances, but certain methods such as personalised phone or postal reminders are manpower intensive [20-22]. With about 1,800 appointments a day in our clinics, the majority of which are actualised without intervention, having such predictions may lead to cost savings by targeting interventions towards patients with higher likelihood of defaulting.

Our analysis concurred with previous studies which showed that long waiting periods, repeat defaulters, and younger age groups are associated with increased likelihood of defaulting [1,10,13]. There are several findings of note that have not previously been reported. We found differences in the odds of attendance amongst different ethnic groups, which may reflect cultural differences that are amenable to interventions. Further studies are needed to explore the reasons for higher failure rates in Malay and Indian patients. More importantly, those who provided a cell phone number had an odds of actualising appointments 6 to 17 times higher than those who did not. This finding may be a conglomeration of various factors. Cell phone ownership may be an indicator of higher socio-economic status, which has been shown to be associated with higher rates of actualisation [10]. The provision of cell phone numbers could also indicate a patient's motivational level to attend appointments. Reasons aside, provision of cell phone numbers is an easily available yet robust predictor for appointment actualisation.

Some variables were less significant predictors than expected. We had expected travel distance to influence appointment failures, but the odds ratios were not as large as other variables. This may be due to convenient transportation and relatively short travel times in a small country like Singapore. Hospitalisation before and during the appointment date also did not contribute significantly, which may signify that hospitalisation itself does not preclude the need to seek treatment for other medical problems.

In the stratified departmental analysis, the effect of predictors, apart from cell phone numbers, was not uniform across departments. For example, the effect of duration from scheduling to appointment varies across specialties. This is to be expected because the duration of symptoms, urgency for treatment, and symptom resolution without treatment are different for conditions consulted at different specialties. The presence of this variation necessitates customised algorithms for individual departments in order for optimal predictions of appointment failure to be made.

There are several limitations to our study. We are uncertain if our findings can be generalised to other settings, as inter-institutional and inter-country differences similar to the observed inter-departmental differences may exist. There may also be differences between time-periods. However, while the predicted probability equation is only relevant for this hospital, the analytic process can be replicated using the methods described, since the study relies only on routinely available administrative data, which can be automatically processed for institutions with computerised appointment systems. Detailed data on failed appointments were unavailable and failed attendances may be reappointed as a new appointment if the patient is contactable. In addition, data before August 2000 is unavailable. Increased data definition may help in increasing the predictive accuracy, but the use of aggregate percentages in this study has produced good results. Our study was also unable to analyse failed appointments by clinical condition and symptoms. Other studies have shown that different clinical conditions and health status may be linked to failed attendances [23,24]. Future studies should include such variables to increase the predictive accuracy, but we note that our methodology already has diagnostic accuracy of more than 80% on the basis of routinely available data alone. This shows that an easily automated and reproducible system can have good predictive ability in spite of not incorporating clinical data, which is not available in most computerised appointment systems.

Our findings can be made operational in several ways. Predictions, based on up-to-date and institutionally relevant data, can be uploaded as automated algorithms into appointment systems. Lists of potential defaulters can then be generated using a desired sensitivity cut-off for targeted interventions to reduce appointment failure. In addition, educational messages can be targeted during prior appointments, based on automated profiling of future failure risk. Another strategy that is commonly used is over-booking to decrease opportunity costs but this can result in increased wait times if overdone. With the forward predictions on the expected appointment failure rate of a future clinic session, over-booking strategies can be optimised.

## Conclusion

Failed appointments result in inefficiencies and economic cost and may interrupt continuity of care. We attempted to address the causes in an outpatient clinic and found that a few key routinely available variables could adequately account for appointment failure. The predicted probability model could predict failures with reasonable accuracy. Administrators can use these techniques to uncover factors in their own clinic deserving of further study. In addition, there is potential for incorporating automated algorithms into information systems to achieve better targeting of interventions, as well as to optimise overbooking strategies.

## Competing interests

The author(s) declare that they have no competing interests.

## Authors' contributions

VJL was involved in all areas including conceiving and designing the study, the data collection, the statistical analysis and writing of the paper. AE was involved in conceiving the study, the data collection, statistical analysis and writing of the paper. MIC was involved in designing the study and writing of the paper. BK involved in conceiving the study and writing of the paper. All authors read and approved the final manuscript.

## Pre-publication history

The pre-publication history for this paper can be accessed here:


